# Cognitive behavioural therapy for the management of inflammatory bowel disease-fatigue with a nested qualitative element: study protocol for a randomised controlled trial

**DOI:** 10.1186/s13063-017-1926-3

**Published:** 2017-05-11

**Authors:** Micol Artom, Wladyslawa Czuber-Dochan, Jackie Sturt, Christine Norton

**Affiliations:** King’s College London, Florence Nightingale Faculty of Nursing & Midwifery, James Clerk Maxwell Building, 57 Waterloo Road, London, SE1 8WA UK

## Abstract

**Background:**

Fatigue is one of the most prevalent and burdensome symptoms for patients with inflammatory bowel disease (IBD). Although fatigue increases during periods of inflammation, for some patients it persists when disease is in remission. Compared to other long-term conditions where fatigue has been extensively researched, optimal management of fatigue in patients with IBD is unknown and fatigue has rarely been the primary outcome in intervention studies. To date, interventions for the management of IBD-fatigue are sparse, have short-term effects and have not been implemented within the existing health system. There is a need to integrate current best evidence across different conditions, patient experience and clinical expertise in order to develop interventions for IBD-fatigue management that are feasible and effective. Modifying an existing intervention for patients with multiple sclerosis, this study aims to assess the feasibility and initial estimates of efficacy of a cognitive behavioural therapy (CBT) intervention for the management of fatigue in patients with IBD.

**Methods:**

The study will be a two-arm pilot randomised controlled trial. Patients will be recruited from one outpatient IBD clinic and randomised individually to either: Group 1 (CBT manual for the management of fatigue, one 60-min session and seven 30-min telephone/Skype sessions with a therapist over an eight-week period); or Group 2 (fatigue information sheet to use without therapist help). Self-reported IBD-fatigue (Inflammatory Bowel Disease-Fatigue Scale) and IBD-quality of life (United Kingdom Inflammatory Bowel Disease Questionnaire) and self-reported disease activity will be collected at baseline, three, six and 12 months post randomisation. Illness perceptions, daytime sleepiness, anxiety and depression explanatory variables will be collected only at three months post randomisation. Clinical and sociodemographic data will be retrieved from the patients’ medical notes. A nested qualitative study will evaluate patient and therapist experience, and healthcare professionals’ perceptions of the intervention.

**Discussion:**

The study will provide evidence of the feasibility and initial estimates of efficacy of a CBT intervention for the management of fatigue in patients with IBD. Quantitative and qualitative findings from the study will contribute to the development and implementation of a large-scale randomised controlled trial assessing the efficacy of CBT interventions for IBD-fatigue.

**Trial registration:**

ISRCTN Registry, ISRCTN17917944. Registered on 2 September 2016.

**Electronic supplementary material:**

The online version of this article (doi:10.1186/s13063-017-1926-3) contains supplementary material, which is available to authorized users.

## Background

Inflammatory bowel disease (IBD) mainly encompasses two related but distinct conditions of the gastrointestinal tract: ulcerative colitis (UC) and Crohn’s disease (CD). UC is characterised by diffuse mucosal inflammation limited to the rectum and colon. CD is characterised by patchy, transmural inflammation, affecting any part of the gastrointestinal tract from the mouth to the anus [1–3]. IBD is a lifelong condition, which frequently presents in adolescence or young adulthood and follows an unpredictable relapsing and remitting course. The prevalence of IBD in the United Kingdom (UK) is about 300,000 with CD and UC affecting 5–10 and 10–20 new patients, respectively, per 100,000 people per year [4].

Patients with IBD are affected by a number of symptoms, undergo lifelong pharmacological treatment and have an increased risk of malignancy [5, 6]. Due to the severity of IBD, psychological distress is also common [7], with prevalence of symptoms of depression and anxiety around 22% and 35%, respectively [8]. As IBD has an early life onset, a chronic nature and does not generally shorten lifespan, addressing how patients deal with their disease is an important aspect of care [9]. The current standard care in IBD treatment is aimed at managing the inflammatory response during flare episodes and maintaining remission, with an emphasis on adhering to a regular medication regime [10]. However, manifestations of IBD cannot be fully accounted for by pathophysiology and the simple targeting of inflammation does not necessarily reduce the symptoms affecting patients the most [11–13]. In line with the international expert consensus of the recent Therapeutic Targets in Inflammatory Bowel Disease (STRIDE) initiative [14] on treatment targets for IBD, it is therefore important to shift from management of IBD aimed solely at achieving endoscopic remission to also evaluating patient reported outcomes (PROs) with the ultimate goal of improving patients’ quality of life (QoL) [15].

Fatigue is a common and predominant concern for patients with IBD, experienced by 44–86% of patients with active disease and 22–41% of patients in remission [16]. Fatigue is a complex, multifactorial and multidimensional phenomenon, which has been described as a ‘persistent overwhelming sense of tiredness, weakness, or exhaustion’ [17], that can be mental, physical or both [18]. Unlike everyday tiredness, fatigue is often unrelieved by sleep or rest [19], can have a substantial negative impact on patients’ QoL [20–23] and it may limit patients in their everyday lives [24]. Although fatigue understandably increases during periods of inflammation, for some patients it persists when disease is in clinical and endoscopic remission [25].

Despite the pervasiveness of fatigue as a chief complaint in IBD patients [23], it is only identified and treated in a relatively small proportion of those affected [26]. The causes of fatigue in IBD are not well understood by either patients [27] or healthcare professionals (HCPs) [28]. Disease activity [22, 29–31], anaemia [32–34] and inflammation [32] have been found to be predictive of fatigue, yet there is a considerable number of IBD patients with no apparent physiological underpinnings for their fatigue [35]. Apart from a consistent relationship with disease activity, IBD-fatigue has been linked to psychosocial factors such as low mood [31, 36, 37] and sleep problems [29, 31, 37, 38]. However, most previous research has focused on the relationship between fatigue and clinical variables. The ways in which clinical variables and potentially modifiable factors interact with each other in fatigue has rarely been explored [39].

The complex aetiology of fatigue [16], its subjective nature [40] and the lack of objective ‘gold standard’ to measure fatigue [41], also add to the challenge of developing suitable and effective management methods. Optimal management of fatigue in patients with IBD is unknown and fatigue has rarely been the primary outcome in intervention studies [42–45]. Pharmacological trials utilising biologic therapy [46–48], thiamine [43] or ferumoxytol [49] have shown potential benefits for fatigue. However, these are contrasted by findings in observational studies showing higher fatigue in patients taking biologic therapy [23, 30, 31] and it is unclear whether these effects are due to a reduction in inflammation or to a direct influence on fatigue-signalling pathways [50]. Despite the known importance of physical activity in IBD-fatigue [31, 51], the only trial [45] examining the effect of advice to increase physical activity provided inconclusive results. A few psychosocial interventions, such as problem-solving, solution focused therapy (SFT) [42, 44] and stress management [52], have shown promising effects which declined over time and studies included only a small number of participants.

Stress management [52] has been compared with conventional medical treatment in 45 patients with CD. Self-directed stress management significantly reduced tiredness post-treatment, at six-month and 12-month follow-up. However, fatigue was measured with a symptom diary rating devised by the authors and not with a validated fatigue scale for the assessment of fatigue in long-term conditions [41]. Moreover, fatigue was not the primary outcome of the intervention, making the mechanisms of change difficult to assess. The efficacy of SFT on fatigue and QoL was evaluated in 98 patients with quiescent IBD [44]. After the intervention and at three-month and six-month follow-up, the SFT group showed a significantly greater reduction in fatigue and improvement of QoL comparable to the care as usual group. Yet, the effect was not maintained at nine months post intervention. It is therefore important to design interventions with longer-term effect of reducing fatigue (with follow-up assessments of a minimum of 12 months), in order to determine whether positive effects of interventions can be sustained over long periods of time, and if not, establish the reasons for loss of response over time [53].

Compared to IBD, where fatigue has not been well described, understood or managed [16], in other long-term conditions fatigue has been extensively researched. Drawing from evidence on fatigue in other chronic conditions with relapsing and remitting trajectory, such as multiple sclerosis (MS) and rheumatoid arthritis (RA), can help to identify the mechanism in which psychosocial and clinical factors interact with each other and contribute to higher levels of fatigue, and identify types of interventions for fatigue management in IBD patients. A few fatigue treatment protocols based on cognitive-behavioural models [54–56], employed in large-scale randomised controlled trials (RCTs) in inflammatory conditions (e.g. MS, RA) have found promising results in fatigue reduction [57–59]. Although varying across conditions and symptoms, cognitive-behavioural models are based on the premise that symptoms are maintained by maladaptive cognitive and behavioural factors [60]. Initially, primary disease factors, such as inflammation, may trigger symptoms of fatigue. The ways in which people react cognitively, emotionally and behaviourally to their fatigue may perpetuate or worsen the symptoms [54]. Consequently, altering cognitions, emotions and behavioural responses in relation to fatigue through cognitive-behavioural therapy (CBT) may improve clinical and psychosocial outcomes [61, 62].

Van Kessel et al. [57] assessed the efficacy of delivering CBT or relaxation training (RT) to 72 patients with MS and found that the CBT group reported significantly greater reductions in fatigue nine months post intervention compared to the RT group. Thomas et al. [62–64] compared six weekly sessions of group-based CBT with current local practice in 164 patients with MS and found statistically significant differences in fatigue severity in favour of the intervention group four months and one year post intervention. Similarly, in a two-arm, parallel RCT in adults with RA, Hewlett et al. [58] compared six weekly sessions and a consolidation session of group CBT with self-management information in a 1-h didactic group session. At 18 weeks from the intervention start, CBT participants reported better scores than control participants for fatigue impact and perceived fatigue severity.

For people with IBD, CBT has been utilised for a variety of outcomes, including relapse reduction [65] and perceived stress [66]. Six systematic reviews have collectively appraised studies of psychological treatment for IBD [67–72] with encouraging results. In the most recent systematic review and meta-analysis [72] of RCTs comparing psychological therapy with a control intervention or a control treatment (14 studies), a significant difference in depression scores and quality of life with psychological therapy was observed at the end of therapy in patients with quiescent disease. However, beneficial effects were lost at the final point of follow-up. When assessing the effect of individual physiological therapies on quality of life, only CBT had any significant beneficial effect. In a systematic review of 17 studies, Goodhand et al. [68] concluded that CBT was effective for mood disorders and improved QoL in patients with IBD. McCombie et al. [71] and Knowles et al. [70] evaluated studies of the main types of psychological treatment for IBD and found that CBT and its variants most commonly contributed to positive outcomes as compared to other psychotherapies (i.e. psychoeducation and problem-solving therapy). CBT consistently resulted in improved psychological distress, but with modest effects in gastrointestinal symptoms these were not sustained over time. However, these findings have to be interpreted in light of limitations, including: time and travel burden for patients attending face-to-face sessions; and high attrition rates and low compliance issues for online interventions [73, 74].

There is a need to integrate current best evidence across different conditions, patient experience and clinical expertise in order to develop interventions for IBD-fatigue management that are feasible and effective [75]. Balancing the insights from research and practice can maximise the likelihood of interventions being feasible and acceptable, and of treatment addressing important issues to patients while overcoming previously identified limitations [76].

There is evidence from RCT interventions in other long-term conditions [57–59] to suggest that modifying maladaptive cognitions and behaviours through CBT could be a viable option for the management of fatigue. Drawing from interventions in other conditions can enhance the process of development of new evidence-based management strategies in IBD without ‘reinventing the wheel’ [77]. Previous quantitative [78, 79] and qualitative studies [80] and systematic reviews [16, 39] showed similarities between the perceived experience of fatigue between different disease groups. Across conditions, fatigue is closely related to relapse and more active disease [81, 82] and similar physical and psychosocial factors appear to be causing and exacerbating fatigue [80]. Additionally, the findings from our recent study [83] indicate that the ways patients perceive, interpret and react to fatigue symptoms in IBD is largely comparable to patients with MS specifically [84]. Patients who have more negative perceptions of fatigue, higher levels of all-or-nothing and avoidance behaviours have significantly greater fatigue scores compared to those with less negative cognitions and behaviours in relation to their fatigue.

Based on these premises, the CBT intervention developed by Van Kessel and colleagues [57] for MS was chosen as the basis of our intervention study for IBD patients. Iterative work with both patients and HCPs working with patients with IBD was then conducted in order to tailor the intervention to IBD patients’ needs following the guidelines on Patient and Public Involvement (PPI) involvement in healthcare [85, 86]. An initial trial has shown promising results with patients with MS [57], has a strong theoretical grounding in a CBT model [87] and has incorporated a valuable and in-depth mediation analysis of processes of change after the trial [88]. The intervention involved eight weekly sessions of 50 min and covered: an introduction to the CBT model of fatigue; activity scheduling; changing unhelpful cognitions surrounding MS and fatigue; sleep hygiene and restoring sleep–wake cycles; managing negative emotions; and the role of social support. In MS, calculated effect sizes for fatigue from baseline to the end of treatment were 3.03 for the CBT group and 1.83 for the RT group, with clinically significant improvements in fatigue. Furthermore, in our study [83], negative perceptions of fatigue and avoidance behaviours were identified in patients with IBD. In MS patients in the trial [88] these same perceptions of fatigue and avoidance behaviours improved significantly more in the CBT than in the RT group. Changing negative perceptions of fatigue mediated the decrease in severity of fatigue [88]. Content of the intervention is described in more detail in Van Kessel et al. [57].

As a result of the challenges inherent in evaluating complex interventions such as CBT, the UK Medical Research Council (MRC) Framework for the development of complex intervention [89] recommends a stepwise approach, with an early piloting phase prior to the design of a large-scale trial. Pilot studies resemble the main study in many respects [90], they are a smaller version of the main study that test whether its components can all work together [91]. They are a requisite initial step in exploring a novel intervention [92] and resemble the main study in many respects, including an initial assessment of the primary outcome [93]. The pilot phase hence ensures that money is not wasted on an expensive trial which produces a null result due to problems with recruitment, retention or delivery of the intervention [94] and that end results are more applicable to real-world settings. It was therefore decided to conduct a pilot study prior to a definitive full-scale effectiveness RCT.

The MRC guidance for process evaluation of complex interventions [95, 96] advocates the potential value of the qualitative research in health interventions. This is part of the growing call to move away from the inappropriate use of pilot trials as hypothesis testing to a greater emphasis on their descriptive, feasibility potential [97]. RCTs are considered the ‘gold standard’ for providing evidence in decision-making in evidence-based practice [98], yet they have been criticised for not providing sufficient evidence that is useful in practice [99]. After the trial, qualitative approaches can help to explore reasons for the findings, examine the appropriateness of the underlying theory and steer researchers towards interventions more likely to be effective in the future [100]. Furthermore, qualitative process evaluation supports understanding and explanation of the processes involved during the implementation of an intervention and its potential integration in everyday practice [101]. To date, only an estimated 3–8% of trials have incorporated qualitative research components [102]. The current pilot trial will hence include a nested qualitative component, with interviews with the IBD patients, the therapist/s delivering the intervention and HCPs working with patients with IBD. The nested qualitative component will strengthen the findings by evaluating perceptions of the current pilot intervention and ultimately enhance the acceptability of the intervention in a large-scale trial.

Research questions: What is the feasibility of a CBT intervention for the management of fatigue in patients with IBD? What are the initial estimates of efficacy of an intervention for the management of fatigue in patients with IBD?

## Methods and design

This study will assess the feasibility and initial estimates of efficacy of a CBT intervention for the management of fatigue in patients with IBD. The pilot intervention will have the objectives to:

- Assess the feasibility of recruiting eligible patients;
- Assess the willingness of participants to be randomised;
- Evaluate the compliance rates to the intervention (Therapist Sessions and Homework Sheets);
- Assess withdrawal and dropout rates during the treatment phase;
- Assess the completion rates of the outcome measures post intervention and at follow-up times;
- Determine the adequate sample size for definitive full-scale effectiveness RCT;
- Obtain initial estimates of efficacy on fatigue and QoL in the CBT intervention group compared to the fatigue Information Sheet group;
- Obtain detailed qualitative feedback from patients, the therapist/s delivering the intervention and HCPs working with patients with IBD on their experience and views of the intervention and areas for improvement in future fatigue interventions.

The pilot trial will not attempt to provide evidence of clinical effectiveness for the CBT intervention in people with IBD. This is in accordance with the recommendations from the National Institute of Healthcare Research (NIHR) guidelines and the Consolidated Standards of Reporting Trials (CONSORT) for the development of pilot studies [91, 103].

### Study design

The study will be a two-arm pilot RCT. Patients will be recruited from the outpatient IBD clinic at a single specialist hospital site and randomised individually, using a 1:1 ratio computerised algorithm. A nested qualitative study will evaluate patient and therapist experience, and HCPs perceptions of the intervention. The study will have a total duration of 22 months with two phases.

### Phase 1: the pilot randomised controlled trial (*n* = 40)

#### Recruitment

A member of the direct care team at the recruitment site will look through electronic and paper medical records, reviewing the inclusion/exclusion criteria, and identify potentially eligible patients attending the IBD outpatient clinic that day. Consecutive potentially eligible patients attending outpatient IBD clinics from January 2017 to June 2017 will be included in the recruitment process. At the end of their appointment a direct member of their care team will ask patients if they would be happy to be approached by a researcher to take part in a research study. If the patient is interested, they will then be directed to the research room. A member of the research team will hand the patients a Patient Information Sheet (PIS) and provide them with a full verbal explanation of the RCT. Patients will be given adequate time to fully comprehend the content of the PIS and will be given the opportunity to ask questions about taking part in the study. Those who indicate a potential interest in the RCT will be screened for full eligibility using the Eligibility Screening Form. If ineligible, the patient will be thanked for their interest, will not be enrolled in the study and no personal details will be recorded.

Eligible patients will then be given at least 48 h to consider their participation in the study and discuss the decision with family, friends and their care team. A member of the research team will then contact them by telephone to answer any additional questions, verify their understanding of what is involved and confirm their interest in study participation. If patients agree to take part in the study, they will be asked to sign the *Patient Consent Forms-RCT* and complete the *Baseline Data A* booklet. A format for written questions for this phase has been developed and piloted with our PPI group to ensure acceptability and ease of understanding of questions to be asked. Participants will be asked to return the signed *Patient Consent Forms-RCT* and the completed questionnaires in the pre-paid stamped addressed envelope provided within seven days of receipt. The returned study documents will be checked for completeness, and the patient will then be entered into the randomisation database for the RCT and told whether they are in Group 1 (CBT manual + therapist support) or Group 2 (Fatigue Information Sheet only). Screening and consenting will continue until the study target sample size (*n* = 40, 20 for each arm) is reached. Hospital consultants will be informed of the patients’ participation in the study via an entry made in the patient’s notes; with patients’ agreement, their General Practitioner (GP) will be informed using *GP Notification Letter*.

#### Randomisation

Consenting participants will be randomised to CBT manual plus therapist support or Fatigue Information Sheet only using a random number generator with a 1:1 ratio in the Statistical Package for the Social Sciences Version 22. All baseline information will be collected prior to randomisation. Participants will be randomised at the individual level. The randomisation sequence will be generated electronically by an independent statistician prior to the commencement of the study. The statistician will have no patient contact. The trial coordinator (blinded until this point) will access the randomisation database to assign patients to the two groups. Indeed, as this is a small pilot study on a limited budget, there are no resources available to provide a blinded data collector. Owning to the nature of the study the participants, the researchers and the therapist will not be blinded to treatment allocation after randomisation. The trial coordinator will be informed of the outcome of the randomisation procedure in order to identify participants who require telephone support calls during the trial. The researcher conducting the qualitative interviews will also be unblinded to ensure that appropriate questions are asked.

#### Group 1 (CBT manual + therapist support)

Group 1 participants will receive a CBT manual for the management of fatigue and have one 60-min session and seven 30-min telephone/Skype sessions with a therapist over an eight-week period. All sessions will be over the telephone or Skype according to patient preference. In the Consent Form, patients will be informed that if they choose Skype, information may not be secure and may be transferred to other countries outside the Trust’s control. During the intervention, participants will have access to all usual care, including the nurse-led helpline.

#### CBT manual development

The CBT manual utilised for the intervention will be a modification of the CBT manual for the management of MS fatigue developed by Van Kessel et al. [57]. The manual contains eight sessions. Despite, the lack of consensus from systematic reviews on the adequate number of therapy sessions to be used by interventions, the number of sessions was modelled on the number of sessions utilised in the RCT by Van Kessel et al. [57], where 100% of the subjections in the CBT group completed the eight-session intervention with large effect sizes. The sessions include: IBD fatigue explained; CBT for IBD fatigue; activity scheduling; improving your sleep; understanding IBD symptoms; changing your thinking; managing stress, determining a sense of control and coping with emotions; social support and preparing for the future, and it has an approximate length of 100 pages. One of the authors (Rona Moss-Morris) of the original CBT manual for MS helped in making the initial changes and refinements to the manual incorporating evidence from the first MS trial [57] and its adaptation into an Internet-based CBT self-management programme for fatigue in MS [104].

In order to make the intervention relevant and acceptable to patients with IBD, we have worked with people with IBD-fatigue, consultant gastroenterologists, IBD-nurse specialists, dieticians and psychologists working with people with IBD. Specifically, Session 1 of the intervention manual ‘IBD-Fatigue Explained’ addresses medical factors causing fatigue which are specific to patients with IBD including inflammation and fatigue and anaemia and fatigue. Furthermore, examples and tasks throughout the intervention manual have been adapted to reflect concerns and issues specific to patients with IBD and not MS. Finally, a medical writer conducted in-depth pre-post readability statistics and performed the required changes to transform the intervention manual into plain English and make the language as clear as possible. Likewise, a graphic designer made the necessary formatting edits to the manual so as to make the design more user-friendly.

#### Therapists

Patients will have telephone support sessions with one of two qualified CBT therapists who have experience in delivering interventions to patients with long-term conditions. The therapist will receive the manual in advance and have the opportunity to discuss its contents and any questions with the research team. The purpose of the telephone/Skype support calls are to promote engagement with the intervention and to support the patient in collaboratively developing goals to work on using the resources and information available to them in the CBT manual. At the start of each telephone call, the therapist will set an agenda with the participant. The first telephone/Skype support call will be scheduled for when the participant will have completed the first session in the CBT manual.

#### Group 2 (Fatigue Information Sheet only)

Group 2 participants will receive the Crohn’s and Colitis UK (CCUK) ‘Fatigue in IBD’ Information Sheet to use without therapist help. CCUK is the UK’s leading charity for patients with CD and UC. CCUK provides patients with free online information sheet and guides to help those affected by IBD. As many people with IBD suffer from fatigue, CCUK, together with our research team (CN, WCD), developed an Information Sheet which explains what fatigue is, what may cause it and possible ways of reducing it.

#### Feasibility and acceptability outcomes

Feasibility of recruiting eligible patients will be evaluated by calculating the proportion of those invited to take part in the intervention that were eligible and then were consented into the trial. The willingness of participants to be randomised will be evaluated by calculating the proportion of those who dropped out of the trial after they have been randomised. Refusal, withdrawal and dropout rates from the study and number of sessions with therapist will be recorded. A post-intervention follow-up questionnaire included in *Outcome Data B* booklet will assess compliance rates to the intervention by asking participants about the number of sessions of the manual they read and time per week spent completing tasks in relation to the intervention. The therapist/s will also record how many sessions the participants have completed. Furthermore, in order to assess compliance to the intervention procedures, if participants are willing to do so, they will be asked to provide the research team with the Homework Sheets completed at the end of each session in the manual. The Homework Sheets will be checked for completion in order to assess compliance with the intervention components.

#### Initial estimates of efficacy outcomes

Prior to randomisation, eligible participants will complete the study baseline measures which are contained in a single questionnaire booklet. The questionnaire booklet contains 20 printed pages. Preliminary piloting has demonstrated that on average it has taken 10–15 min to complete and our patient representatives have commented that this is not unduly burdensome. Three months post randomisation, the Outcome Questionnaire booklets will be sent by post. Patients who were allocated to the CBT intervention and will be asked further questions about their experience of completing the intervention in order to assess their compliance with the intervention, preferences, acceptability and satisfaction with the intervention. Patients who were allocated to Fatigue Information Sheet only, will be asked about their experience of reading the Fatigue Information Sheet. A postal reminder will be sent to non-responders two and four weeks after the seven-day response period has ended, utilising the Reminder Letter and/or a telephone call*.* Six and 12 months post randomisation, two more outcome booklets will be sent respectively, with two postal reminders and/or telephone calls for non-responders after two and four weeks. The booklets will only contain the primary outcome measures IBD-fatigue, QoL UK questionnaire and Disease Activity Indexes (DAIs) specific to the condition.

#### Twelve months post randomisation

Patients in the Fatigue Information Sheet only group (Group 2) will be offered the CBT manual for managing fatigue in IBD. No therapist support will be offered alongside the manual.

Nested qualitative study (*n* = 7 patients + both therapist/s + approximately three HCPs)

To better understand patient perspectives on the intervention, participants consenting to the RCT might be invited for interview. The interview sub-sample will be purposively selected to include both genders, a range of ages, both IBD diagnoses and those successful and not successful in showing an initial improvement in IBD-fatigue. Purposive sampling has the potential to provide richer, more relevant and diverse data pertinent to the research question [105, 106]. Participants will be asked about the process of recruitment and randomisation, their experience of the intervention itself, their reasons for dropping out or not completing (where appropriate) and areas for improvement in the design of future fatigue interventions for patients with IBD. Patients will receive information about the interviews in the *Patient Information Sheet – RCT and Interviews*. Using the *Patient Consent Form – Interviews*, separate informed consent will be sought for the face-to-face/telephone/Skype, semi-structured interviews with a purposive sample of approximately seven participants (about one-third of the participants in Group 1). Interviews will be conducted by a researcher not involved in the delivery of the intervention and have a duration of 30–60 min.

The nested qualitative study will be conducted after the three-month follow-up quantitative data collection point. The choice of qualitative data collection at three months allows for minimisation of recall bias, patient burden and confounding of participants in the study. The short time period between the intervention and the interviews, ensures that participants will find it easier to recall their experience of the intervention. Indeed, involvement in qualitative data collection as part of an RCT may in some way influence the participant’s experience of treatment [107]. However, the gap in time between the interviews and the next follow-up data collection point (six months post randomisation) limits the potential for participants’ outcome responses to be influenced by their participation in the interviews. Furthermore, time gap in time between interviews and follow-up questionnaire completion reduces potential patient burden, while still ensuring continuity to the study.

The therapists supporting patients during the intervention will be interviewed to understand their experience of delivering the intervention and to inform future adaptation and delivery of the intervention in clinical practice. HCPs working with patients with patients with IBD at the study site will be interviewed to obtain their views on the intervention and its possible implementation within existing IBD service at a roll out stage. Approximately three HCPs will be interviewed. Informed consent for the interview will be secured using the *Staff Consent Form – Interviews.*

#### Qualitative analysis

Interviews will be conducted by a researcher who will not be involved in the delivery of the intervention. Interviews will be digitally audio-recorded, anonymised and transcribed verbatim by a professional transcriber. Original audio files and file transcripts will be stored on a secure server at King’s College London (KCL) in a password protected file. The transcriber will delete his/her copy of each audio file once transcription is complete. Data will be analysed using thematic analysis [108] and, if appropriate, NVivo11 software for data management. Analysis begins with a coding framework, where key concepts emerging from the transcripts will be mapped. Additional themes emerging are added to the coding framework. The final framework is agreed, then applied to all transcripts. An iterative process will confirm a final coding framework before analysis. Two researchers will code all transcripts independently and then compare and refine resulting codes and themes in discussion. The emergent themes will form the basis of analytical interpretation. Process evaluation data will be analysed separately from outcome data in order to avoid bias in interpretation [109].

### Study outcome measures

For feasibility and acceptability outcomes, please refer to Study outcome measures. The primary and secondary outcome measures will be recorded at baseline, three, six and 12 months post randomisation. The primary outcome measure will be utilised to assess initial estimates efficacy of the intervention in the CBT intervention group compared to the Fatigue Information Sheet group. All outcome measures have been validated for self-completion.

### Primary outcome measure

#### IBD-Fatigue Scale (IBD-F)

The IBD-F [110] aims to assess IBD-specific fatigue. The first two sections of the questionnaire, five questions assessing frequency and severity of fatigue and 30 questions rating the experience and impact of fatigue, will be utilised in the study. Higher scores indicate higher fatigue and higher impact of fatigue. Initial validation of the measure suggested that the questionnaire had good face and content validity, acceptable to excellent test–retest stability and a high degree of internal consistency [110]. Both sections of the IBD-F have been found to be significantly correlated with other widely utilised fatigue scales [22]. The scale was developed by conducting in-depth interviews with participants with IBD, in order to gain insight into their experience and ultimately to represent issues of specific importance to people with IBD fatigue.

### Secondary outcome measures

#### United Kingdom Inflammatory Bowel Disease Questionnaire (UK IBDQ)

The UK IBDQ [111] is the British version of the McMaster IBDQ [112]. It has 32 items, each scored in the range of 1–4, with a summary score between 30 and 120. A low score indicates poor quality of life. Initial findings have supported the reliability, validity, reproducibility and responsiveness of the UK version of the questionnaire. The questionnaire has been found acceptable to patients in the UK. It enhances the precision of some of the questions in the McMaster IBDQ, improves the readability of the questionnaire, removes items that do not provide useful information and simplifies the response categories. The IBDQ was developed with patients with IBD, it therefore reflects the concerns of the patients themselves about the impact of their disease on their life-style and quality of life. The IBDQ is recommended for use in healthcare evaluation to assess the effect of interventions for IBD on QoL [113]. Members of our patient representatives group voted 15:2 in favour of the UK version when compared with the original version, stating ‘choices more straightforward’.

### Explanatory variables

#### Disease activity indexes

The Harvey Bradshaw Index (HBI) [114] and the Simple Clinical Colitis Activity Index (SCCAI) [115] will be utilised to measure disease activity for CD and UC patients, respectively. The HBI and SCCAI will be recorded at baseline, three, six and 12 months post randomisation. All other explanatory variables will only be recorded at baseline and three months post randomisation.

#### Brief Illness Perceptions Questionnaire (BIPQ)

The BIPQ [116] uses a single-item scale approach to assess illness perceptions. It is a shorter version of the original Illness Perception Questionnaire (IPQ) [117] which is utilised to assess five dimensions within a cognitive representation of illness. The brief version consists of nine items: five of the items assess cognitive illness representations (consequences, timeline, personal control, treatment control and identity), two of them assess emotional representation (concern and emotions) and one item assesses illness comprehensibly. Each item (e.g. ‘How concerned are you about your fatigue?’) is rated using a response scale of 0–10; in which higher scores represent more threatening views of fatigue. The psychometric properties of this measure have been assessed using samples from several illness groups including IBD [118] and chronic obstructive pulmonary disease [119].

#### Epworth Sleepiness Scales (ESS)

The ESS [120] is utilised to measure a participant’s level of daytime sleepiness. From a clinical point of view this is relevant in that it helps to determine the presence of pathology or simply predict whether sleep onset is likely to occur at inappropriate times [121]. The questionnaire asks participants to rate their chance of falling asleep or dozing on a scale of 0–3 in eight soporific situations, ranging from ‘Lying down to rest in the afternoon when circumstances permit’ to ‘in a car while stopped for a few minutes in traffic’. A total score of 0–24 is determined, with values over 10–11 indicating abnormal or pathological sleepiness. Given its ease of use and cost-effectiveness, the ESS in now one of the most widely used tools for the assessment of sleepiness [122].

#### Seven-item Generalised Anxiety Disorder Scale (GAD7)

The GAD7 [123] asks participants how often during the last two weeks they have been bothered by each of the seven core symptoms of generalised anxiety disorder. Response options are ‘not at all’, ‘several days’, ‘more than half the days’ and ‘nearly every day’, scored as 0, 1, 2 and 3, respectively. It has a minimum possible score of 0 and a maximum possible score of 21. The GAD7 has been utilised in studies assessing anxiety severity in diverse conditions, including: patients with eating disorders [124]; multiple sclerosis [125]; and cardiovascular disease [126]. The GAD7 is used as an outcome measure for CBT for anxiety in the UK Improving Access to Psychological Therapies (IAPT) programme [127].

#### Nine-item Patient Health Questionnaire (PHQ9)

The PHQ9 [128] is based on the diagnostic criteria for major depressive disorders in the Diagnostic and Statistical Manual, Fourth Edition (DSM-IV). The questionnaire contains nine items, which are scored from 0 (not at all) to 3 (nearly every day), according to the frequency of their experience over the previous two-week period, with a total score in the range of 0–27. The PHQ9 has been found to be a reliable and valid measure [129] and it has been previously validated in gastroenterological patients [130]. The PHQ9 is used as an outcome measure for CBT for depression in the IAPT programme.

### Sociodemographic and clinical data

Sociodemographic and clinical data about participants will be collected at baseline in order to better characterise the sample, make the results of the RCT more clinically relevant and appropriately adjusting statistical data analysis. Sociodemographic data collected will include: age; gender; marital status; education status; employment status; and living arrangements. Clinical data will include: IBD diagnosis; co-morbidities; latest measurement of faecal calprotectin concentration; IBD-related medications (name, dose and frequency); length of time since diagnosis (years, months); number of IBD-related surgeries; smoking status (current smoker, ex-smoker, never smoked); exercise status (> or < 30 min of aerobic exercise per week); haemoglobin; ferritin; serum albumin; C-reactive protein (CRP); platelets count; vitamin B12; and folate. These clinical data are routinely collected as part of standard care in patients with IBD at these sites. If the patient does not attend the service at follow-up, it is unknown whether there will be updated clinical data for patients. However, due to potential patient burden and lack of funding, no additional blood tests will be conducted for the patients, even if recent clinical data are not present in the hospital patient records. One value for each clinical marker will be collected if it refers to up to three months prior to or after the completion of the baseline questionnaires.

### Participant entry

Patient interviewees will be selected from those recruited for the RCT, who will have been evaluated prior to enrolment. The *Eligibility Screening Form* will be used to conduct pre-registration evaluation of participants for the RCT.

### Inclusion criteria

- Patients who are currently experiencing fatigue (self-reported)
- Proof of diagnosis of IBD (record of diagnostic endoscopy in patient clinical notes); patients without this test will not be included
- Aged 18 years and over
- No elevated inflammatory markers or other clinical features of active disease

### Exclusion criteria

- Patients without a record of diagnostic endoscopy in their clinical notes
- Elevated inflammatory markers or other clinical features of active disease
- Course of CBT for any reason in the last year
- Currently enrolled in another trial involving a novel pharmacological intervention
- Current or planned pregnancy (pregnant women have been found to be significantly more affected by fatigue and sleep disturbances [131])
- Inability to give informed consent (for example, due to reduced mental capacity)
- Insufficient command of written and spoken English to understand study documents or procedures

### Statistics and data analysis

Due to the pilot design of the study a power calculation was not required. Data collected in the pilot study will be used to generate information for sample size calculations for a definitive full-scale effectiveness RCT. Based on similar pilot studies, a sample of 40 patients (20 per arm) was deemed large enough to provide useful information about the aspects that are being assessed for feasibility. It is recognised that the study may not be powered to detect meaningful differences in clinically important endpoints [91].

Patients will be recruited from an IBD outpatient clinic in London, UK, which is representative of the target study population of patients experiencing IBD-fatigue. The sample was based on the same inclusion/exclusion criteria that would be used in a future definitive full-scale effectiveness RCT. At the study site, a total of approximately 100–150 patients attend four IBD outpatient clinics each week. Assuming around 40% recruitment of eligible patients based on our earlier studies recruiting from this population [83], discussions with the clinical team and recruitment rates for intervention for fatigue in MS [57], our target aim of 40 patients should be reached within the six-month baseline data collection period. Taking into account an attrition rate based on prior research [132] of about 20% for each follow-up, we will aim to achieve a minimum sample size of 40 at baseline, in order to suitably assess outcomes at follow-up.

Primary and secondary measures at baseline along with recruitment rates, telephone session attendance, time spent on the intervention, Homework Sheet completion rates and withdrawal from intervention rates will be presented as means and standard deviations for approximately normally distributed continuous variables, medians and interquartile ranges for non-normally distributed variables, frequencies and percentages for categorical variables. Initial estimates of treatment effect on primary and secondary outcomes at the follow-up assessments will use an intention-to-treat (ITT) framework, implemented using a regression model, adjusting for baseline values of the outcome, sociodemographic and clinical outcomes. ITT analysis will compare the primary and secondary outcome measures at three months between the two randomised groups. ITT analysis will compare only primary outcome measures at six and 12 months between the two randomised groups. Where participants wish to withdraw from the intervention, we will attempt to retain them in the data collection, unless they express a wish to be withdrawn completely.

As this is a pilot study, it is not intended that the study is powered to detect significant differences on the primary or secondary outcome measures. Qualitative data will also be recorded. Protocol conforms to Standard Protocol Items: Recommendations for Interventional Trials (SPIRIT). See Additional files 1 and 2 at the end of the manuscript for SPIRIT Figure and Checklist.

## Discussion

This is the first RCT to utilise CBT for the management of fatigue in patients with IBD. To date, psychosocial interventions utilising problem-solving, solution focused therapy (SFT; 42, 44) and stress management [52] have shown promising effects. However, their effects declined over time [44] and fatigue was not always the primary outcome of the intervention [52]. The study will provide evidence of the feasibility and initial estimates of efficacy of a CBT intervention for the management of fatigue in patients with IBD. Quantitative and qualitative findings from the pilot study will contribute to the development and implementation of a subsequent large-scale RCT assessing the efficacy of CBT interventions for IBD-fatigue.

One of the limitations of the pilot trial is the small number of participants which will impact on the statistical strength of the study. However, the lack of sufficient data about the feasibility and efficacy of CBT for the management of fatigue in this population necessitates the undertaking of an initial pilot upon this issue.

### Trial status

Patient recruitment for the study will begin in January 2017 and is expected to continue for six months in total.

## Additional files

Additional file 1: SPIRIT Checklist. (DOC 104 kb)

Additional file 2: SPIRIT Figure. (DOC 61 kb)

## Abbreviations

CBT: Cognitive behavioural therapy
CD: Crohn’s disease
CONSORT: Consolidated Standards of Reporting Trials
CRP: C-reactive protein
CV: Curriculum vitae
DSM: Diagnostic and Statistical Manual
ECCO: European Crohn’s & Colitis Organisation
GAD: Generalised Anxiety Disorder
GCP: Good clinical practice
GP: General practitioner
HCP: Healthcare professional
IBD: Inflammatory bowel disease
ITT: Intention to treat
MS: Multiple sclerosis
NHS: National Health Service
NIHR: National Institute of Healthcare Research
NRES: National Research Ethics Service
PPI: Patient public involvement
PRO: Patient reported outcome
RA: Rheumatoid arthritis
RCT: Randomised controlled trial
REC: Research Ethics Committee
RT: Relaxation therapy
SFT: Solution focused therapy
UC: Ulcerative colitis
UK: United Kingdom
