# Supplementary material for: Cognitive behavioural therapy for the management of inflammatory bowel disease-fatigue with a nested qualitative element: study protocol for a randomised controlled trial
Source: Trials. 2017 May 11;18:213. doi: 10.1186/s13063-017-1926-3 (PMC5425996; doi:10.1186/s13063-017-1926-3)
Supplement: Supplementary file 1 — SPIRIT Checklist. (DOC 104 kb) [file 13063_2017_1926_MOESM1_ESM.doc]

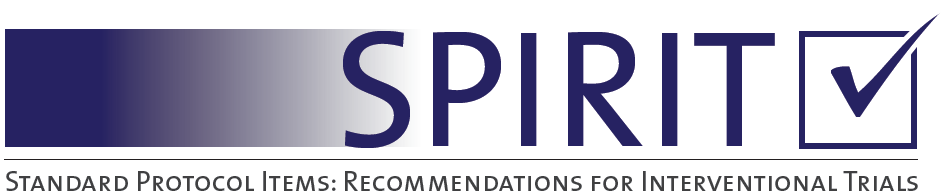


SPIRIT 2013 Checklist: Recommended items to address in a clinical trial protocol and related documents*

| Section/item | ItemNo | Description |
| --- | --- | --- |
| **Administrative information** | | |
| Title | 1 | Page 1 |
| Trial registration | 2a | Page 2 |
| Protocol version | 3 | Page 2 |
| Funding | 4 | Page 19 |
| Roles and responsibilities | 5a | Page 19 |
|  | 5b | NA |
|  | 5c | NA |
|  | 5d | NA |
| Introduction |  |  |
| Background and rationale | 6a | Pages 4-8 |
|  | 6b | Page 10 |
| Objectives | 7 | Page 8 |
| Trial design | 8 | Page 9 |
| Methods: Participants, interventions, and outcomes | | |
| Study setting | 9 | Page 9 |
| Eligibility criteria | 10 | Page 16 |
| Interventions | 11a | Page 10 |
|  | 11b | NA |
|  | 11c | NA |
|  | 11d | Page 16 |
| Outcomes | 12 | Pages 11-16 |
| Participant timeline | 13 | Pages 8-12 |
| Sample size | 14 | Page 17 |
| Recruitment | 15 | Page 17 |
| **Methods: Assignment of interventions (for controlled trials)** | | |
| Allocation: |  |  |
| Sequence generation | 16a | Page 9 |
| Allocation concealment mechanism | 16b | Page 9 |
| Implementation | 16c | Page 9 |
| Blinding (masking) | 17a | Page 10 |
|  | 17b | NA |
| **Methods: Data collection, management, and analysis** | | |
| Data collection methods | 18a | Pages 13-16 |
|  | 18b | Page 12 |
| Data management | 19 | Page 17 |
| Statistical methods | 20a | Page 17 |
|  | 20b | Page 17 |
|  | 20c | Page 17 |
| **Methods: Monitoring** | | |
| Data monitoring | 21a | NA |
|  | 21b | NA |
| Harms | 22 | NA |
| Auditing | 23 | NA |
| Ethics and dissemination | | |
| Research ethics approval | 24 | Page 19 |
| Protocol amendments | 25 | NA |
| Consent or assent | 26a | Page 12 |
|  | 26b | NA |
| Confidentiality | 27 | Page 13 |
| Declaration of interests | 28 | NA |
| Access to data | 29 | NA |
| Ancillary and post-trial care | 30 | NA |
| Dissemination policy | 31a | NA |
|  | 31b | NA |
|  | 31c | NA |

*It is strongly recommended that this checklist be read in conjunction with the SPIRIT 2013 Explanation & Elaboration for important clarification on the items. Amendments to the protocol should be tracked and dated. The SPIRIT checklist is copyrighted by the SPIRIT Group under the Creative Commons “[Attribution-NonCommercial-NoDerivs 3.0 Unported](http://www.creativecommons.org/licenses/by-nc-nd/3.0/)” license.
