# Supplementary material for: Cognitive behavioural therapy for the management of inflammatory bowel disease-fatigue with a nested qualitative element: study protocol for a randomised controlled trial
Source: Trials. 2017 May 11;18:213. doi: 10.1186/s13063-017-1926-3 (PMC5425996; doi:10.1186/s13063-017-1926-3)
Supplement: Supplementary file 2 — SPIRIT Figure. (DOC 61 kb) [file 13063_2017_1926_MOESM2_ESM.doc]

Figure 1. SPIRIT schedule of enrolment, interventions, and assessments.

| **TIMEPOINT** | **2017** | | | | | | | | | | | | **2018** | | | | | | | |
| --- | --- | --- | --- | --- | --- | --- | --- | --- | --- | --- | --- | --- | --- | --- | --- | --- | --- | --- | --- | --- |
|  | 1 | 2 | 3 | 4 | 5 | 6 | 7 | 8 | 9 | 10 | 11 | 12 | 13 | 14 | 15 | 16 | 17 | 18 | 19 | 20 |
| **ENROLMENT:** |  | | | | | | | | | | | | | | | | | | | |
| Eligibility screening |  |  |  |  |  |  |  |  |  |  |  |  |  |  |  |  |  |  |  |  |
| Informed consent |  |  |  |  |  |  |  |  |  |  |  |  |  |  |  |  |  |  |  |  |
| Randomisation |  |  |  |  |  |  |  |  |  |  |  |  |  |  |  |  |  |  |  |  |
| **INTERVENTIONS:** |  | | | | | | | | | | | | | | | | | | | |
| Group 1 intervention |  |  |  |  |  |  |  |  |  |  |  |  |  |  |  |  |  |  |  |  |
| Group 2 intervention |  |  |  |  |  |  |  |  |  |  |  |  |  |  |  |  |  |  |  |  |
| **ASSESSMENTS:** |  | | | | | | | | | | | | | | | | | | | |
| Measures at baseline |  |  |  |  |  |  |  |  |  |  |  |  |  |  |  |  |  |  |  |  |
| 3 month follow-up outcome measures |  |  |  |  |  |  |  |  |  |  |  |  |  |  |  |  |  |  |  |  |
| Qualitative interviews |  |  |  |  |  |  |  |  |  |  |  |  |  |  |  |  |  |  |  |  |
| 6 month follow-up outcome measures |  |  |  |  |  |  |  |  |  |  |  |  |  |  |  |  |  |  |  |  |
| 12 month follow-up outcome measures |  |  |  |  |  |  |  |  |  |  |  |  |  |  |  |  |  |  |  |  |
| Intervention manual given to all participants |  |  |  |  |  |  |  |  |  |  |  |  |  |  |  |  |  |  |  | **X** |
